# Using an Extreme Lateral Interbody Fusion (XLIF) in Revising Failed Transforaminal Lumbar Interbody Fusion (TLIF) With Exchange of Cage

**DOI:** 10.7759/cureus.14123

**Published:** 2021-03-26

**Authors:** Anwar M Al-Rabiah, Zahraa I Alghafli, Ibrahim Almazrua

**Affiliations:** 1 Department of Orthopaedics, King Faisal Specialist Hospital and Research Centre, Riyadh, SAU; 2 Department of Surgery, King Salman Hospital, Riyadh, SAU

**Keywords:** pseudoarthrosis, lateral interbody fusion, revision surgery, interbody cage migration, percutaneous pedicle screw

## Abstract

Minimally invasive techniques have gained popularity in spine surgery in recent years. Extreme lateral interbody fusion (XLIF) is one of these techniques. The rapid increase in the use of this approach in either primary or revision surgeries is related to its several advantages including less operative time, less blood loss and reduced length of hospital stay with fast recovery. We report a case of a failed transforaminal lumbar interbody fusion (TLIF) in L4-L5 level, one year after the primary procedure with persistent pain due to failed fusion. Underwent revision, by using XLIF with the removal of old cage and exchange with new large cage. Revision of failed interbody fusion can be achieved through anterior, posterior or lateral approach. The decision to proceed with either method depends on several factors, including previous surgeries, fibrosis and risk of neurovascular injury and surgeon’s preference. XLIF approach should be considered in revision surgeries of failed interbody fusion. As it can provide several advantages compared to anterior or posterior approaches, in terms of better fusion rates and lower risk of neurovascular injuries by avoiding the use of the previous passage.

## Introduction

Extreme lateral interbody fusion (XLIF) is a minimally invasive technique that approaches the spine through the retroperitoneal approach, using muscle splitting through the psoas. It can allow access to the spine's anterior aspect from the T6 level to L5 [1,2]. It has been reported that it has fewer complications and morbidity rates compared to anterior lumbar interbody fusion (ALIF) and posterior lumbar interbody fusion (PLIF) [3]. These complications include pseudarthrosis, graft dislodgement, and neurologic injury [3].

Failure of transforaminal lumbar interbody fusion (TLIF) due to pseudoarthrosis or unexpected retropulsion of interbody cage can result in a relapse of pre-operative symptoms. These findings might necessitate the need for revision surgery. Traditionally, both anterior and posterior approaches were used to retrieve the cage and revise failed TLIF [4]. XLIF technique used in revision spinal surgeries has been reported on a few occasions [5,6]. Along with adjacent level syndrome, indications of XLIF include degenerative disc disease with instability, recurrent disc herniation, and post-laminectomy syndrome [5,6].

The rapid increase in its popularity is related to its several advantages, including shorter operative time, less blood loss, smaller wounds, reduced length of hospital stay, and fast recovery [3]. Besides, there is a lower risk of neural injuries, and vascular injuries can be achieved using the XLIF approach compared to posterior or anterior approaches, respectively [7-9]. Moreover, others reported higher rates of fusion observed with XLIF, together with stability, due to its privilege of allowing larger cages [5]. However, it is not without any complication. Some of the reported include iliopsoas weakness, anterior thigh numbness, quadriceps weakness, radiculopathy, and iliopsoas hematoma [10,11].

We report our surgical experience using the extreme lateral approach to remove an unfused cage, which was done through the TLIF approach and exchanging it with a larger cage with percutaneous pedicle screw fixation.

## Case presentation

The patient is a 42-year-old Arabic male smoker with, known case of diabetes mellitus. Referred to our clinic due to a history of persistent low back pain with bilateral radiculopathy. In the outside hospital, he underwent L4-L5 decompression and TLIF with pedicle screw fixation in 2018. Two weeks later, they took him to remove right-side pedicle screws as there was a breach medially.

With MRI report at that time showing posterior broad-based disc herniation at L4-L5 level indenting the ventral aspect of the thecal sac and both neural exit foramina, more on the right side, with mild spondylolisthesis. 

The pain subsided after surgery two months ago, where he started again to complain about severe low back pain radiating more to the right side. It was gradually increasing over time, not preceded by any form of trauma or heavy lifting. Aggravated by walking and relieved partially with pain medications and rest. No numbness or paresthesia reported by the patient and no constitutional symptoms. Negative history of bowel or bladder dysfunction or other cauda equina symptoms such as motor weakness, sensory loss, or saddle anesthesia.

**Figure 1:**
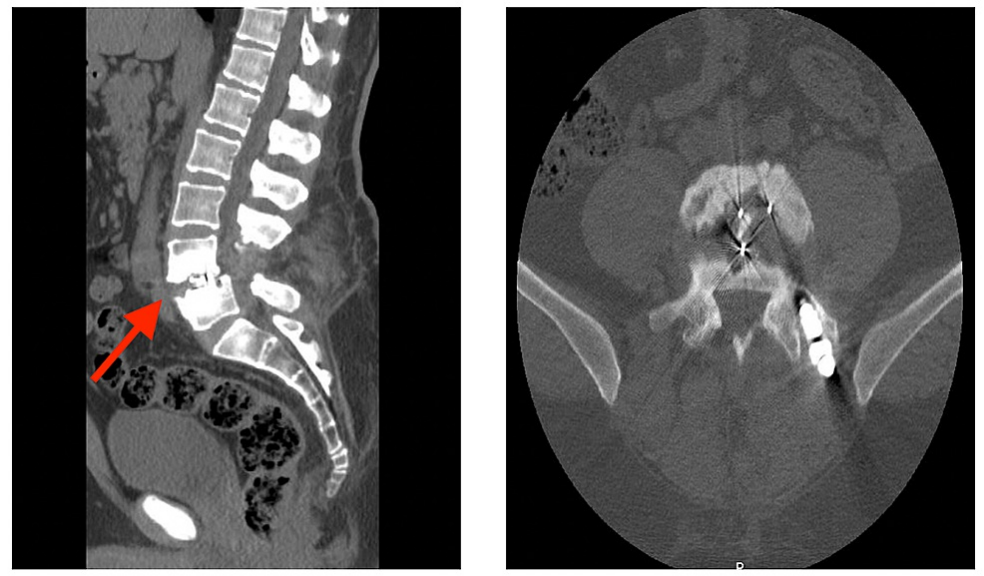
Pre-revision CT scan. CT scan reported as sclerotic L4-L5 opposed vertebral endplates (red arrow) with irregular outlines and no fusion can be appreciated.

On examination, no obvious deformity can be appreciated, with a scar in the lumbar area from previous surgery. No hotness or tenderness was noted, and the range of motion was from 75 degrees of flexion to 15 degrees of extension. The distal neurovascular exam was unremarkable. The Straight-leg-raise test was positive at 40 degrees bilaterally. Investigations in the form of infection workup, CT scan, and MRI were done. CBC, ESR, and CRP were normal. CT scan reported as sclerotic L4-L5 opposed vertebral endplates with irregular outlines and no fusion can be appreciated (Figure 1).

**Figure 2:**
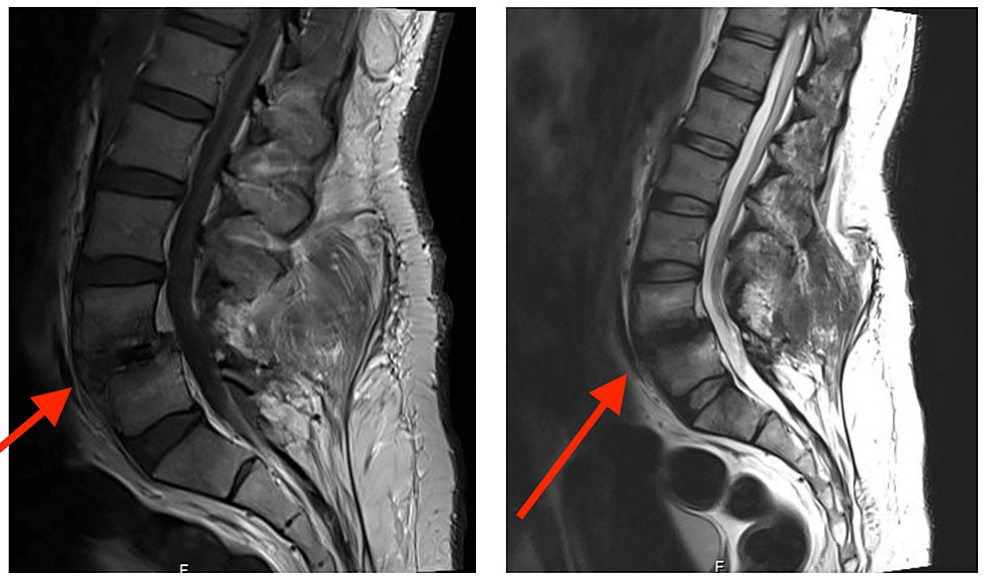
pre-revision MRI of lumbar spine showing post-operative changes and mild anterior translation of L4 over L5 with associated bilateral facet arthropathy (red arrows), with combined lytic and degenerative 1st-degree anterolisthesis.

MRI showed mild anterior translation of L4 over L5 with associated bilateral facet arthropathy, with combined post-operative lytic and degenerative 1st-degree anterolisthesis. In addition to diffuse posterior disc bulge effacing the epidural fat and the theca and encroaching upon the corresponding lateral neural exit foramina without any clear abscess formation (Figure 2).

**Figure 3:**
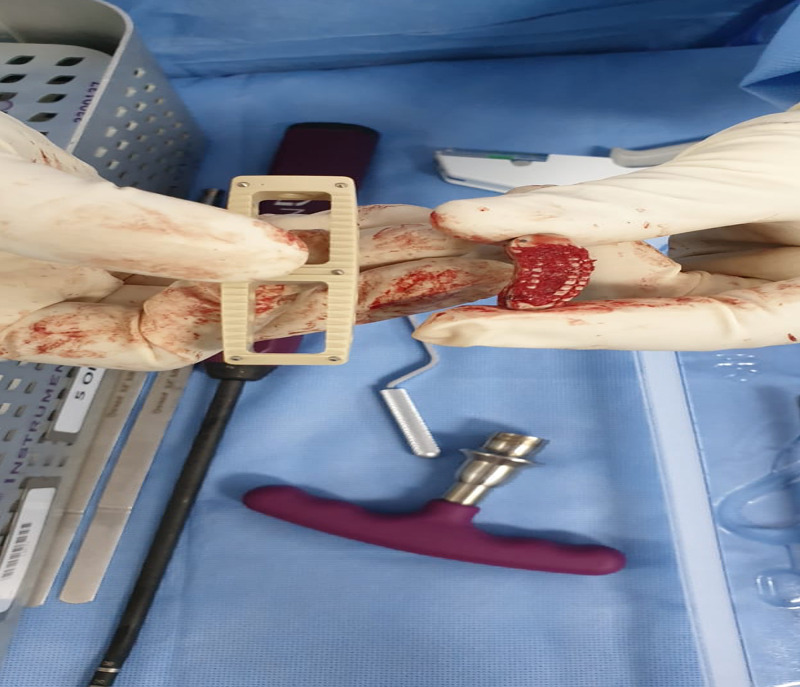
Intra-operative image showing the removed cage.

We took him for revision surgery in the form of removal of TLIF cage from L4-L5 with XLIF and percutaneous pedicle screw fixation for the right side. The standard XLIF approach was utilized; we reached the lateral disc space through the lateral approach. Then the interbody cage was detached from the L4-L5 endplates using Cobb and osteotome (Figure 3).

A gap was made between the endplates and the interbody cage to make them mobile and loose. Once it was clearly loose by the addition of curettage for the surrounding scar tissues, a successful attempt was made to remove the interbody cage with the pituitary. After that, percutaneous pedicle screws were inserted on the right side only for L4-L5 vertebrae.

**Figure 4:**
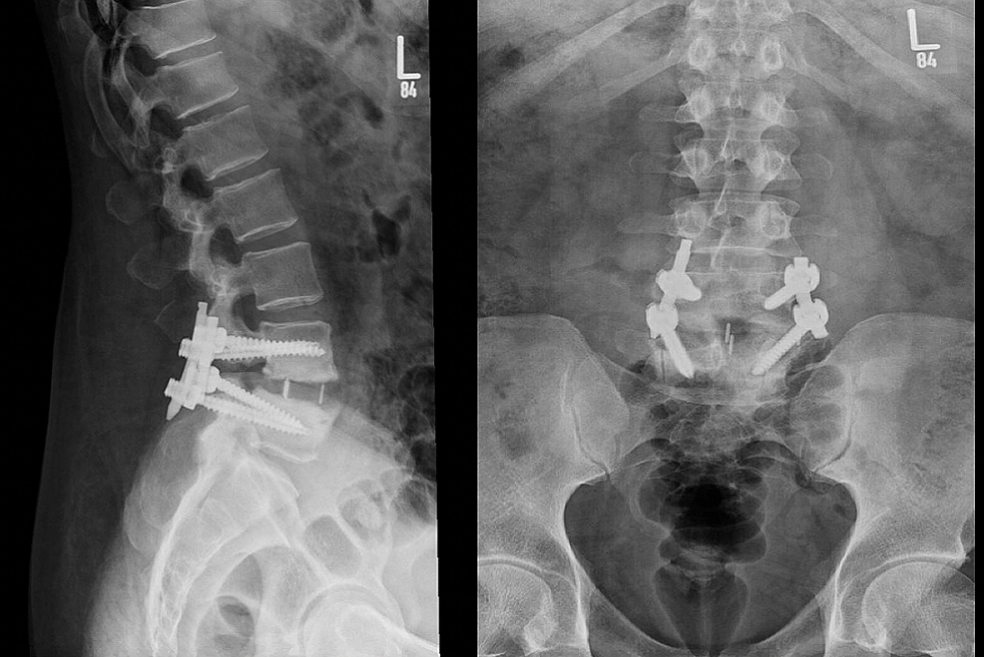
Lateral and AP X-rays of lumbar spine one-month post-revision.

One-month postoperatively, the patient is seen in the clinic with improvement in pain and radiculopathy. Post-operative X-rays were satisfactory (Figure 4).

## Discussion

TLIF is widely used in lumbar spinal fusion as it provides circumferential fusion through a pedicle screw placement approach using techniques such as pedicle screw plate fixation, cage compression, and placement and nerve root protection [12]. The patient has disc herniation at L4-L5 level indenting the ventral aspect of the thecal sac and both neural exit foramina, more on the right side, with mild spondylolisthesis. The disc herniation is of the protrusion type observed from the MRI, which led to severe back pain radiating more to the right side where the disc herniation had been spotted [13]. The pain gradually increased overtime nor preceded by any form of trauma or heavy lifting since, during the TLIF, the compression done to the patient's pedicle screw after insertion of the cage causes an upward movement of the superior face, which in this case compressed the patient's exiting nerve root [6]. This is because the rods are pre-curved during the TLIF to achieve the desired lordosis, which, if there is a complication, leads to, in this case, aggravation while walking.

CT and MRI should be used to examine post-operative contralateral led pain since the first examination showed no obvious deformity with just a scar on the patient's lumber area, which was from a previous surgery [14]. However, the CT and MRI were significant as they showed mild anterior translation of L4 over L5 with associated faced arthropathy, with a combined post-operative lytic and degenerative 1st-degree anterolisthesis. The examination showed no tenderness or hotness, with a positive straight leg test. Anterolisthesis showed an abnormal alignment of bones in the patient's spine, which affected his lower back leading meaning that the upper vertebra slipped in front, causing pain to the patient. The CT and MRI revealed a failed TLIF due to pseudoarthrosis, or unexpected retropulsion of interbody cage, resulting in a relapse of pre-operative symptoms [12,13]. To diffuse posterior disc bulge effacing the epidural fat and the theca and encroaching upon the corresponding lateral neural exit foramina without any clear abscess formation and a sclerotic L4-L5 opposed vertebral endplates with irregular outlines and no fusion can be appreciated.

XLIF completely retains the anterior and posterior longitudinal ligament, which was reached using the lateral approach, which allowed access to lateral disc space. However, the anterior approach in removal on interbody cage poses similar risks as the ones gotten from using TLIF cage and complications such as fibrosis. Using this approach, the interbody cage was detached from the L4-L5 endplates using Cobb and osteotome. The surgeon accessed the intervertebral disc space and fused the low back using a lateral approach from the side rather than accessing the anterior or posterior. With the gap made between the endplates and the interbody cage to allow mobility as it was loose due to the addition of curettage for the surrounding scar tissues but through a successful attempt, the interbody cage with pituitary was removed and after that percutaneous pedicle screws were inserted on the right side only for L4-L5 vertebrae as it was the side with complications. 

The patient came back to the clinic one-month post operativity. There was an improvement in pain and radiculopathy, meaning that the revision surgery using XLIF was successful. The percutaneous pedicle screw on the right side had been successfully fixed. This proves that there is a lower risk of neural injuries and vascular injury treatments using the XLIF approach than the TLIF approach, which had previously caused complications to the patient's pedicle screw fixation. There is a higher success rate of fusion using XLIF as it allows the privilege of insertion of larger cages. XLIF has recently become a minimally invasive technique used to approach the spine using different lateral and retroperitoneal approaches, having various advantages.

## Conclusions

In this study, the approaches discussed are TLIF and XLIF, where XLIF is used to revise complications caused by the TLIF approach to treat persistent low back pain with radiculopathy. TLIF is associated with many complications, as seen in other interbody fusion methods. The use of CT and MRI helps in accessing and examining interbody fusion rates and utilizing XLIF, which has lower risks to correct the complication incurred.
